# A Novel Approach to Dream Content Analysis Reveals Links Between Learning-Related Dream Incorporation and Cognitive Abilities

**DOI:** 10.3389/fpsyg.2018.01398

**Published:** 2018-08-06

**Authors:** Stuart M. Fogel, Laura B. Ray, Valya Sergeeva, Joseph De Koninck, Adrian M. Owen

**Affiliations:** ^1^The Brain and Mind Institute, Western University, London, ON, Canada; ^2^School of Psychology, University of Ottawa, Ottawa, ON, Canada; ^3^The Royal’s Institute of Mental Health Research, Ottawa, ON, Canada; ^4^Brain and Mind Research Institute, University of Ottawa, Ottawa, ON, Canada

**Keywords:** sleep, dreams, intelligence, memory, hypnagogic, cognitive ability

## Abstract

Can dreams reveal insight into our cognitive abilities and aptitudes (i.e., “human intelligence”)? The relationship between dream production and trait-like cognitive abilities is the foundation of several long-standing theories on the neurocognitive and cognitive-psychological basis of dreaming. However, direct experimental evidence is sparse and remains contentious. On the other hand, recent research has provided compelling evidence demonstrating a link between dream content and new learning, suggesting that dreams reflect memory processing during sleep. It remains to be investigated whether the extent of learning-related dream incorporation (i.e., the semantic similarity between waking experiences and dream content) is related to inter-individual differences in cognitive abilities. The relationship between pre–post sleep memory performance improvements and learning-related dream incorporation was investigated (*N* = 24) to determine if this relationship could be explained by inter-individual differences in intellectual abilities (e.g., reasoning, short term memory (STM), and verbal abilities). The extent of dream incorporation using a novel and objective method of dream content analysis, employed a computational linguistic approach to measure the semantic relatedness between verbal reports describing the experience on a spatial (e.g., maze navigation) or a motor memory task (e.g., tennis simulator) with subsequent hypnagogic reverie dream reports and waking “daydream” reports, obtained during a daytime nap opportunity. Consistent with previous studies, the extent to which something new was learned was related (*r* = 0.47) to how richly these novel experiences were incorporated into the content of dreams. This was significant for early (the first 4 dream reports) but not late dreams (the last 4 dream reports). Notably, here, we show for the first time that the extent of this incorporation for early dreams was related (*r* = 0.41) to inter-individual differences in reasoning abilities. On the other hand, late dream incorporation was related (*r* = 0.46) to inter-individual differences in verbal abilities. There was no relationship between performance improvements and intellectual abilities, and thus, inter-individual differences in cognitive abilities did not mediate the relationship between performance improvements and dream incorporation; suggesting a direct relationship between reasoning abilities and dream incorporation. This study provides the first evidence that learning-related dream production is related to inter-individual differences in cognitive abilities.

## Introduction

The question “why do we dream?” has been a topic of debate throughout recorded history and, although the topic is widely studied, the answer remains highly contentious (Blagrove and Akehurst, 2000; Blagrove and Pace-schott, 2010) and still unresolved. Even more fundamental is the question of how dreams are constructed. It has been suggested that the nature of the cognitive qualities of dreams is an emergent property of an individual’s intellectual strengths and weaknesses. Foulkes (1982, 1985) proposed that dreams manifest from fundamental cognitive functions, such as information processing, problem solving, and planning. Others have sought to understand dreams through identifying putative trait-like, psychometrically assessed correlates of dream production (e.g., memory capacity, personality traits, sleep characteristics, electroencephalography, and psychopathology). Surprisingly, experimental evidence to support a link between cognitive abilities and dream content remains sparse and inconsistent, thus, few conclusive links have been identified (Blagrove and Akehurst, 2000; Blagrove and Pace-schott, 2010). There are several reasons this could be the case. For example, previous work has focused exclusively on the dreams of rapid eye movement (REM) sleep, possibly overlooking any relationship between cognitive abilities and dreams during non-REM sleep. In comparison to REM sleep, relatively little is known about mental activity during non-REM sleep, and in particular, hypnagogic reverie. There is now recent and compelling evidence to suggest that novel waking-life experiences are incorporated into the content of non-REM dreams, particularly with the formation of new memories (Wamsley and Stickgold, 2010; Oudiette and Paller, 2013; Wamsley, 2014). Thus, suggesting that non-REM dream content can reflect the reprocessing of newly learned material. Yet, it remains to be investigated whether the extent of incorporation of newly formed memories into dream content is related to trait-like cognitive abilities. Such a link would demonstrate that dreams can serve as a window into our cognitive strengths or weaknesses, and help us understand the process of dream formation.

There is a large body of evidence suggesting that one of the functions of sleep is for memory consolidation, as reflected by performance improvements (e.g., from training to retest) over an intervening period of sleep (Maquet, 2001; Stickgold and Walker, 2005; Rasch and Born, 2013; Peigneux et al., 2015). More specifically, sleep has been shown to benefit memory for facts, places, and events [i.e., “declarative memory” (Plihal and Born, 1997; Smith, 2001; Gais et al., 2002; Gais and Born, 2004; Schabus et al., 2004; Clemens et al., 2005; Ellenbogen et al., 2006; Fogel et al., 2007b; Payne et al., 2012)], as well as for skills [i.e., “procedural memory” (Nader and Smith, 2003; Fogel and Smith, 2006; Fogel et al., 2007b, 2015; Barakat et al., 2011)]. More specifically, recent evidence from human neuroimaging studies suggest that after new learning, there is physiological reactivation of brain areas recruited during learning (Peigneux et al., 2003, 2006; Bergmann et al., 2011; Antony et al., 2012; Oudiette and Paller, 2013; Oudiette et al., 2013; Schönauer et al., 2014; Fogel et al., 2017). This reactivation of newly acquired memory traces is thought to underlie the process of sleep-related memory consolidation. In addition, single cell recordings in animal studies have shown that there is not only a reactivation of the same brain structures recruited during learning, but during sleep, there is a physiological replaying of the neural representation formed during waking exploration (Skaggs and McNaughton, 1996; Louie and Wilson, 2001; Lansink et al., 2009). In addition, recent converging evidence from humans suggest that behavioral (Oudiette et al., 2011; Uguccioni et al., 2013) and neuronal replay occurs after new learning during subsequent sleep (Buzsaki, 1989; Skaggs and McNaughton, 1996; Louie and Wilson, 2001; Girardeau et al., 2009). This reactivation and replay of newly acquired memories is reflected in the content of our dreams (Stickgold et al., 2000; Wamsley et al., 2010a,b; Kusse et al., 2012; Wamsley, 2014). Furthermore, sleep as compared to an equivalent period of wake, serves to support memory consolidation (Walker, 2005), and the process of consolidation is reflected in non-REM hypnagogic reverie (Wamsley et al., 2010a). However, relatively little is known about whether this is also reflected in the content of waking thought during mind wandering, i.e., “daydreaming.” The characteristics of dreams as compared to daydream content differ; the later containing more references to preoccupations of waking life. Compared with daydreams (van Rijn et al., 2018), non-REM dreams during a daytime nap are less emotionally intense, have fewer sensory experiences, whereas as compared to daydreams, REM dreams are more bizarre with heightened sensory experiences (Carr and Nielsen, 2015), and are a time of increased mind wandering (Fox et al., 2013). However, the incorporation of learning-related content of daydreams vs. dreams remains to be directly compared. Taken together, these studies suggest that dream incorporation reflects memory consolidation processes related to the reactivation and replaying of neuronal events that took place during learning.

The notion that daytime experiences and newly acquired memories are incorporated into the content of dreams is not new. However, several methodological hurdles have presented a challenge to the objective, scientific investigation of dreams. For example: (1) only certain types of experiences (e.g., engaging, emotional, and autobiographical) are robustly incorporated into dreams (Stickgold et al., 2000; Malinowski and Horton, 2014a,b), (2) the type and timing of sleep when dream reports are collected is important for identifying learning-related incorporation (Nielsen et al., 2004; van Rijn et al., 2015), (3) the objective quantification of dream content, until very recently (Amini et al., 2011; Horikawa et al., 2013; Wong et al., 2016) has been limited to subjective assessment of verbal dream reports, and (4) the measurement of the semantic relationship between waking experiences and verbal dream reports (i.e., incorporation) has only been subjectively assessed by comparing behavior to dream reports, as opposed to comparing verbal reports of the learning experience to verbal reports of the subsequent dream experience (Wamsley et al., 2010a,b).

As mentioned above, only certain types of experiences are incorporated into dreams. Early studies investigating the influence of daytime experiences on dream content (Foulkes and Rechtschaffen, 1964; Witkin and Lewis, 1965; Cohen, 1971; De Koninck and Koulack, 1975; Goodenough et al., 1975), used passive and less immersive stimuli such as images and films. Surprisingly, these studies did not identify clear evidence of dream incorporation. It was subsequently found that dream content could be more easily manipulated and detected with the use of novel, immersive, and impactful experiences (De Koninck and Koulack, 1975; Koulack et al., 1985; Corsi-Cabrera et al., 1986; De Koninck et al., 1988, 1990). An important milestone in understanding the phenomena of dream incorporation came from Stickgold et al. (2000) where participants played a variation of the highly engaging video game ‘Tetris’ before sleep. They found that both normal individuals and amnesiacs had similar dream reports, directly incorporating elements of the game into their dreams, even though the amnesiacs did not recall playing the game. Similar findings come from other highly engaging or emotionally arousing tasks such as downhill skiing arcade games (Wamsley et al., 2010a), navigation of a virtual 3D environment (Wamsley et al., 2010b; Solomonova et al., 2015) and stressful/emotionally arousing situations (De Koninck and Koulack, 1975; Koulack et al., 1985; Malinowski and Horton, 2014a). Thus, not all daytime experiences are robustly incorporated, or easily identified in post-learning dream content. Consistent with previous studies, here, highly engaging tasks were employed to maximize the likelihood of identifying dream incorporation.

Not only does the type of experience impact dream incorporation, but the type and timing of the sleep where the dream reports are collected also influence the nature of the reported dream content. While REM sleep is most commonly associated with dreaming (Aserinsky and Kleitman, 1953; Dement and Kleitman, 1957), it is now becoming increasingly clear that some form of dreaming exists in all stages of sleep (Foulkes, 1962; Palagini et al., 2004; Suzuki et al., 2004; McNamara et al., 2010; Oudiette et al., 2012); suggesting that sleep without dreams is akin to wake without thought. For example, hypnagogic reverie during the lighter stages of non-REM sleep (e.g., stages 1 and 2 sleep), traditionally thought not to contain dreams, have been found to contain vivid dreamlike content (Foulkes and Vogel, 1965; Vogel et al., 1972; Vogel, 1991) and most recently, rich incorporations of daytime experiences (Wamsley et al., 2010a,b). Furthermore, several important factors have been identified that can be leveraged to facilitate the study of dreams in non-REM sleep (Vogel, 1991; Nielsen, 2000; Nielsen et al., 2004; Wamsley et al., 2010a), including: (1) allowing only a short delay between the daytime experience and the dream report (e.g., reports collected “early” vs. “late” in the sleep episode), (2) by collecting dream reports after only short bouts of sleep (e.g., short ≤15 s vs. longer ≥45–75 s, or >120 s), and (3) collecting reports from the lighter stages of non-REM sleep (e.g., stage 1 and following the first indications of stage 2 sleep) as compared to deeper slow wave sleep (e.g., SWS). Thus, collecting early dream reports, following short bouts of light non-REM sleep, maximizes the chances of obtaining robust, frequent and direct learning-related dream incorporations (Nielsen, 2017).

Not only does sleep serve to support sleep-related memory consolidation, there is also accumulating evidence suggesting that trait-like inter-individual differences in the characteristics of non-REM sleep (e.g., sleep spindles) relate to trait-like cognitive abilities (Schabus et al., 2006; Bódizs et al., 2008; Fogel and Smith, 2011; Geiger et al., 2011; Gruber et al., 2013; Hoedlmoser et al., 2014; Ujma et al., 2014; Fang et al., 2017). Specifically, this research has found that spindles are highly correlated with reasoning and problem-solving skills (e.g., the ability to employ logic and identify complex patterns; Schabus et al., 2006; Fogel et al., 2007a; Fogel and Smith, 2011; Fang et al., 2017). Given that spindles have also been identified as indices of reactivation of recently acquired information (Bergmann et al., 2011; Fogel et al., 2014), it is probable that this spindle-related reprocessing during non-REM sleep, may be reflected in the content of dreams. However, it remains to be explored whether the extent of learning-related dream incorporation is correlated with inter-individual differences in intellectual abilities. The investigation of the relationship between the extent of dream incorporation following new learning and intellectual abilities may serve as a means to disentangle the links between memory consolidation, dreaming, and cognitive abilities.

Here, we propose to ask the question: are inter-individual differences in the extent of the incorporation of newly learned experiences into dreams associated with waking intellectual abilities? A mediation model was used to test whether the relationship between learning and the incorporation of daytime experiences into dreams can be explained by inter-individual differences in cognitive ability. We predicted that: (1) the extent of memory consolidation will be related to dream incorporation, particularly for early dream reports, when learning-related dream incorporations are more direct (Wamsley et al., 2010a), (2) learning-related dream incorporation will be positively correlated with cognitive abilities, such as reasoning abilities (which have been linked to spindles during non-REM sleep), (3) this relationship may also be stronger during early dream reports when incorporations are more robust, and (4) given that sleep as compared to an equivalent period of wake, serves to support memory consolidation (Walker, 2005), and the process of consolidation is reflected in non-REM hypnagogic reverie (Wamsley et al., 2010a), dream imagery will have greater learning-related incorporation than wake imagery. Finally, (5) a mediation analysis was planned to test whether the relationship between the extent of performance improvements (from training to retest, representing the extent of memory consolidation) and dream incorporation were mediated by inter-individual differences in cognitive abilities.

This study will help to provide insight into the nature of dream formation as it relates to cognitive abilities, such as the capacity for reasoning, problem solving, the use of logic (i.e., “fluid intelligence”) or the application of existing knowledge (i.e., “verbal intelligence”) thus supporting Foulkes’ notion of a close link between cognitive capacity and the level of sophistication in dream production.

## Materials and Methods

### Ethics Statement

All participants were given a letter of information, gave written informed consent prior to participation and were financially compensated for their participation. This study was carried out in accordance with the recommendations of Tri-Council Policy Statement for the Ethical Conduct for Research Involving Humans. This research was approved by the Western University Health Science Research Ethics Board, London, ON, Canada.

### Procedure

**FIGURE 1:**
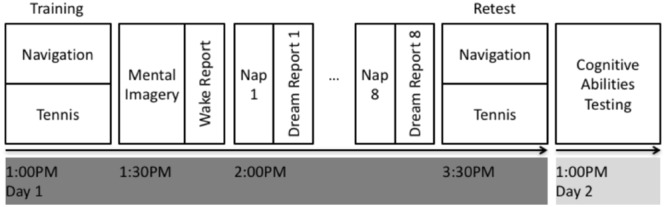
Experimental design. On day 1, subjects performed either the spatial navigation or tennis task for approximately 30 min. They were then asked to mentally rehearse their experience and subsequently provide a detailed wake verbal report of the mental rehearsal. Subjects then underwent PSG monitoring while attempting to fall asleep. Subjects were awoken periodically after signs of light non-REM sleep to provide a dream report or after an equivalent interval of wakefulness to provide a daydream report. Subjects were then retested on the same task after the nap. Finally, at the completion of the experimental protocol, subjects completed the CBS Trials test battery the next day.

See **Figure 1** for an overview of the experimental design. All participants were initially screened to verify that they met the inclusion criteria (see section “Participants and Screening” for details). Each participant underwent two, in-laboratory PSG recordings including an initial acclimatization and screening nap, followed one week later by the experimental nap. For each afternoon, the participant would arrive at the sleep laboratory at 12:00 PM where they were prepared for PSG recording (see section “Polysomnographic Recordings” for details). Following this, on the experimental afternoon, participants were trained on either the spatial navigation task (*N* = 12; see section “Spatial Navigation Task” for details) or the tennis task (*N* = 12; see section “Tennis Task” for details). Participants were then asked to close their eyes and mentally rehearse the task that they had just performed for 30 s, followed by 30 s of rest, where they imagined themselves singing the “alphabet” song. This mental rehearsal and rest alternated a total of 10 times. The rest condition was used to avoid continuing to rehearse the task, reduce mental fatigue, or mind wandering by the participants by having them all imagine themselves singing a commonly known song. Following the mental rehearsal sessions, participants verbally provided a “wake report” describing in detail the mental rehearsal of the task (see section “Verbal Reports” for details). Next, participants were given a 90-minute daytime nap opportunity with PSG monitoring and recording. During this nap, participants were monitored by an RPSGT and woken up after brief periods (>10 s) of non-REM sleep or wake and asked to provide “dream reports” or “daydream reports” (see section “Verbal Reports” for details). Each subject included in the data analysis had a minimum of *N* = 8 dream reports, and *N* = 2 daydream reports. All verbal reports were recorded via audio recording and subsequently transcribed. The extent of incorporation between the wake report and each dream report or daydream report was quantified using a novel approach (See section “Verbal Report Analysis” for details). Following the nap opportunity, participants were retested on the same task as before. Testing on the Cambridge Brain Sciences (CBS) Trials (see section “Cognitive Abilities Testing” for details) took place between the hours of 9:00 AM and 9:00 PM the following day at their optimal time of day, after completion of the experimental protocol so as not to influence the sleep or dream content during the experimental sessions.

### Participants and Screening

A total of 24 healthy young adults (20 female; mean age = 23.3 ± 4.0 years; age range: 20 to 35 years) participated in the study. Sample sizes were determined based on previous studies, and power calculated, where possible, using G^∗^Power for Mac version 3.1 (Faul et al., 2007, 2009). A recent study by our group using the same cognitive tests as the current study (Fang et al., 2017) found robust associations in a sample size of *N* = 24, replicating previous findings in smaller samples (Fogel and Smith, 2006; Fogel et al., 2007a) (n.b., based on power calculation for correlation with α (two-tailed) = 0.05, β = 0.20, effect size = 0.56, *N* = 22 required). To our knowledge, only one study has employed a similar approach to dream content analysis using WordNet (Horikawa et al., 2013), in as few as *N* = 3 individuals; albeit it should be noted they employed a very different statistical approach to the current study. Similar protocols to the present study (Wamsley et al., 2010a) have detected robust dream learning-related incorporation in *N* = 16 participants (based on power calculation for paired *t*-test with α (2-tailed) = 0.05, β = 0.20, effect size = 3.3, *N* = 4 required). Thus, similar to previous reports, a sample size of *N* = 24 was considered to be adequate to detect the cognitive correlations, and *N* = 12 (per task condition) to detect behavioral, cognitive and dream content effects.

Potential participants underwent an initial telephone screening interview and were excluded if they reported they were not in good health, left-handed, considered themselves to be a poor sleeper, had an irregular sleep schedule (slept outside of 10:00 PM to 9:00 AM), had a body mass index >30, were diagnosed with a sleep disorder, worked as a shift worker, took medication known to interfere with sleep, or had a history of psychiatric or mental illness, head injury, or seizure. To be included, interested participants had to score <10 on the Beck Depression Inventory (Beck et al., 1974) and the Beck Anxiety Inventory (Beck et al., 1988), and have no indications of a sleep disorder based on the Sleep Disorders Questionnaire (Douglass et al., 1994).

In addition to these criteria, participants underwent a 90-minute polysomnographic (PSG) recording that served as an acclimatization and sleep disorder screening nap from approximately 1:00 PM to 2:30 PM. The screening nap included electroencephalogram (EEG; from scalp locations Fz, Cz, Pz, and Oz), electrooculogram (EOG; from the left and right outer canthus of the eye), electromyogram (EMG; from the submental chin muscles and anterior tibialis muscle of each leg), respiration (via thorax and abdomen respiratory effort belts), electrocardiogram (ECG; from below each clavicle), and blood oxygen saturation (via a finger probe placed on the index finger of the right hand). Screening nap recordings were manually scored by a single, expert Registered Polysomnographic Technologist (RPSGT) in accordance with clinical scoring guidelines established by the American Academy of Sleep Medicine (Iber et al., 2007). Participants were excluded from further participation in the study if the results of their screening nap revealed greater than 5 respiratory events per hour of sleep or greater than 10 periodic leg movements per hour of sleep.

To verify that participants maintained a regular sleep schedule for the duration of their participation in the study, they were also asked to wear an ‘Actiwatch’ (Philips Respironics Inc., Andover, MA, United States; a wrist-worn accelerometer, to measure sleep-wake-related limb movements) and to complete a log of their daily activities and sleep habits. Participants were excluded from further participation in the study if the actigraphy data or activity and sleep log identified non-compliance with the instructions to maintain a regular sleep-schedule prior to in-laboratory testing.

### Polysomnographic Recordings

Embla Titanium (Natus, Pleasanton, CA, United States) PSG systems were used to perform in-laboratory sleep recordings. Physiological data were recorded at a sampling rate of 512 Hz, with a high pass filter = 0.15 Hz. EEG (from scalp locations: F3, Fz, F4, C3, Cz, C4, P3, Pz, P4, and Oz), EOG (from the left and right outer canthus of the eye), and EMG (from the submental chin muscles) recordings were taken using gold-plated electrodes applied to the skin. Each EEG and EOG channel was recorded and re-referenced offline to an average mastoid reference (M1 and M2). The EMG channel was recorded as a bipolar derivation. Sleep stages were visually scored in 10-second epochs by a single, expert RPSGT in accordance with standard sleep stage scoring criteria (Iber et al., 2007) using RemLogic analysis software (Natus, Pleasanton, CA, United States).

### Behavioral Testing

#### Spatial Navigation Task

A spatial navigation task (Sutton et al., 2014) was used to train and then retest a sub-group of the participants (spatial navigation condition, *N* = 12) before and after a daytime nap opportunity. This task was chosen because the neural correlates of mental imagery for similar tasks have been previously characterized (Owen et al., 2006; Monti et al., 2010; Cruse et al., 2011) and is similar to previous studies investigating the role of sleep (Peigneux et al., 2004; Orban et al., 2006) and dreams (Wamsley et al., 2010b) for spatial learning using 3D virtual environments. In-house software was developed using a modified version of the 3D first person video game, “Team Fortress” (Valve Corporation, Bellevue, WA, United States), for PC. Participants navigated through a 3D environment meant to resemble an Italian village in first person view mode. The virtual environment consisted of six distinct start and goal spatial locations (see Sutton et al., 2014 for details) including a coffee shop terrace, a restroom entrance, a derelict vehicle, bikes on a bike rack, a fruit market, and an Italian flag painted on a wall of a courtyard. For each trial, participants began navigating the maze from one of the start locations and were instructed to navigate to a predetermined goal location (e.g., “find the coffee shop”). The start and goal locations were pseudo-randomly determined for each trial. Participants were instructed to use the keyboard keys W, A, S, and D to navigate forward, left, back, and right, respectively. As well, the left and right arrow keys rotated their point of view of the environment leftward or rightward (but not up or down, in order to restrict the degrees of freedom in movement to simplify the demands required to navigate the environment). Participants were instructed that the objective of the task was to navigate through the environment as quickly as they could, using the shortest route possible to find the pre-determined goal location. Participants completed a total of 30 trials in the training session and 6 trials in the retest session. The number of trials was determined so that the training session would last approximately 30 min in total. Performance was measured in terms of speed (i.e., distance per time taken from the start to the goal location). This measure excluded backtracking away from the target. Offline performance improvements from training to retest with an intervening nap period, were measured as the mean difference in speed from the end of the training session (mean of last two trials) to the start of the retest session (mean of first two trials).

#### Tennis Task

In order to ensure that the extent of the incorporation was specific to the waking experiences of the participants, and not some epiphenomena of similarity between individuals’ reports, another learning experience distinct from spatial navigation was employed. Grand Slam Tennis (Electronic Arts, Redwood City, CA, United States) for the Nintendo (Kyoto, Japan) Wii video game console (with Wii tennis racquet remote) was used to train and then retest another group of the participants (Tennis condition, *N* = 12) before and after a daytime nap opportunity. This task was chosen as the neural correlates of mental imagery for similar tasks have been previously characterized (Owen et al., 2006; Monti et al., 2010; Cruse et al., 2011) and is similar to previous studies investigating the role of sleep (Peigneux et al., 2004; Orban et al., 2006) and dreams (Wamsley et al., 2010b) in immersive game simulations. The experimenter demonstrated the correct position for the participant to stand (e.g., 2 m from the 2.1 m × 1 m image projected on the wall of the testing room with their feet 0.3 m apart from one another), the appropriate grip for holding the “tennis racquet” (e.g., the tennis racquet-shaped motion sensitive game controller), and the manner to serve and swing the racquet. The participants were instructed “to hit the ball over the net and into the opponent’s court in such a way that the opponent is not able to play a good return.” Each testing session followed the normal rules of the game of tennis. In the training session, participants played a match of 20 games. After the nap opportunity, the retest session consisted of a match of five games. In both the training and retest session, participants were always serving (i.e., service did not alternate between participant and opponent). Each game consisted of a series of rallies and was completed when one of the players won four points (i.e., “game point”). Note that in the case of a tie (i.e., “deuce”), the game was won when either the participant or the opponent won two rallies in a row, for a total of five points won. The number of training games was determined so that the play time of the session, similar to the spatial navigation task, would last approximately 30 min in total. Performance for each game was measured in terms of points won per number of rallies played, thus reflecting the efficiency of their win per game. Offline performance improvements from training to retest with an intervening nap period, were measured as the mean difference in points won per rally from the end of the training session (mean of last two trials) to the start of the retest session (mean of first two trials).

### Verbal Reports

#### Verbal Report Collection

**FIGURE 2:**
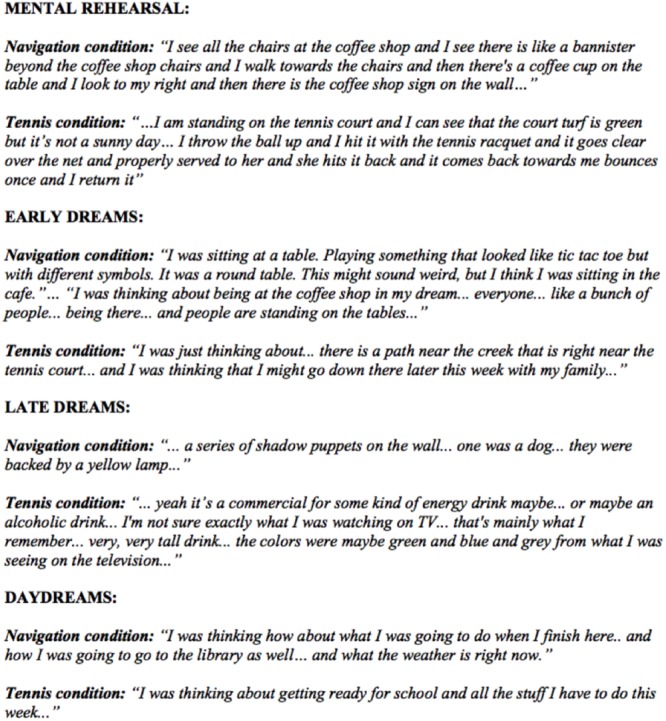
Excerpts from representative examples of wake mental rehearsal, early, late, and daydream reports. Note that in these representative examples, that early dreams contain direct incorporations from the daytime experience of navigating to the goal destinations of the virtual maze (e.g., the café), or the objects in the environment (e.g., a round table), and in another example, mention a tennis court. By contrast, late dreams appear to have incorporations from the lab environment (e.g., references to screens). A description of the lab environment was included in the wake reports to account for non-task specific, but laboratory context-related incorporations. In contrast, the daydream tended to contain preoccupations of the day, rather than incorporations of past task or lab environment elements.

In order to allow participants the opportunity to practice verbally reporting their dreams prior to in-laboratory testing, participants were required to create an audio recording of their verbal dream reports upon awakening each morning for 1 week prior to the training day. On the training day, three distinct types of verbal reports were collected including: (1) a mental rehearsal “wake report,” (2) “dream reports,” and (3) “daydream reports.” The wake report was obtained after a 10-minute mental rehearsal session of the task that they were previously trained on (see section “Behavioral Testing”). Participants were instructed to provide a descriptive verbal report of the task-related mental imagery from the rehearsal sessions. This was also combined with a verbal report describing the lab environment in which they were trained on the task. The “dream reports” and “daydream reports” were obtained throughout the 90-minute daytime nap opportunity. During this nap opportunity, participant’s polysomnographic recordings were visually monitored continuously by an expert polysomnographic technologist and woken up before entering stage 2 sleep, to collect eight dream reports (following non-REM) interspersed throughout the nap opportunity, and as many daydream reports (following wake) as possible. Specifically, participants were allowed to get least 10 s of stage 1, and woken up immediately upon the first signs of stage 2 sleep (indicated by the presence of the first sleep spindle or k-complex). Participants were then asked to provide a dream report by verbally describing, in as much detail as possible, “*what was just going through your mind*” while asleep. The first four dream reports were categorized as “early” and the last 4 dream reports were considered “late” dreams. The “daydream reports” were also collected during the 90 min nap opportunity, however, they were collected after a minimum of 10 s of wake, indicated by visual inspection of the ongoing polysomnographic recording, while the subject was attempting to fall back to sleep, but still awake. It should be noted that there were not enough daydream reports available to divide into early and late, however, we had no a-priori hypotheses from the literature to make such a comparison. A minimum of at least one dream report was collected prior to collecting the daydream report. See **Figure 2** for representative dream examples collected in the different conditions (e.g., early, late, and daydreams).

#### Verbal Report Analysis

**FIGURE 3:**
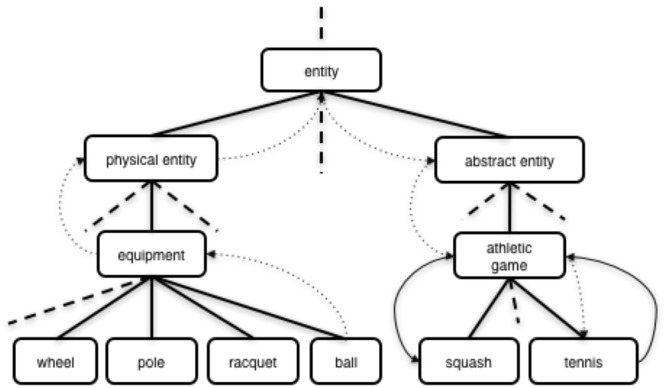
A highly simplified conceptual representation of the WordNet hierarchy. Examples of the shortest path between the more closely related synsets “tennis” and “squash” (solid arrows) versus the path between the more distantly related synsets “ball” and “tennis” (dotted arrows) in a simplified WordNet hierarchy.

Advancements from natural language processing and computational linguistic approaches, in combination with large databases of the English language (e.g., WordNet, a database of semantically disambiguated word senses), can provide a means to objectively interpret verbal dream reports, and also be applied to quantify the extent of dream incorporation. Dream incorporation was measured as the average degree of semantic similarity between the wake report, and: (1) the dream reports (i.e., the reports obtained following periods of sleep), and (2) the daydream reports (i.e., the reports obtained during periods of wake while attempting to fall asleep). Semantic similarity between these sets of verbal reports was computed from WordNet 3.0 (Miller, 1995; Fellbaum, 1998) using tools included in the Natural Language Toolkit (NLTK) for Python (Perkins, 2010). WordNet is a publicly available lexical database of the English language. The WordNet database is a manually curated collection of 155,287 words. WordNet is structured such that each word is grouped into 117,659 sets of synonyms, or “synsets” (i.e., an interconnected hierarchy of groups of synonyms and their brief definitions). Much like a dictionary, each word is represented in terms of its part of speech (e.g., noun, adjective, verb, adverb), meaning (i.e., senses) for polysemous words, and a brief definition (i.e., gloss) using one or more examples. Each word can be a member of more than one synset. In addition, much like a thesaurus, WordNet contains semantic relations (e.g., synonyms, antonyms, parent-child relations or “hyponyms,” and part-of relations or “meronyms”). This information is organized into a hierarchical structure that can be represented as a network (i.e., hypernym tree) of words contained within synsets (see **Figure 3** for an example of a highly simplified conceptual representation of the WordNet structure illustrating the semantic distance between the synsets “tennis” and “squash,” and between the more distantly related synsets “ball” and “tennis”). By representing synsets in a hypernym tree, the semantic distance (i.e., synset similarity) can be calculated using Wu–Palmer Similarity (Wu and Palmer, 1994), a scoring method to compute semantic similarity based on how similar word senses are to one another and where synsets occur relative to one another in the hypernym tree, with scores ranging from 0 (no semantic relationship) to 100 (completely synonymous). The Wu-Palmer method available in the NLTK is advantageous as it can measure similarity between different parts of speech and can account for part-of relations (Wu and Palmer, 1994; Warin, 2004; Petrakis et al., 2006). The Wu–Palmer Similarity computes shortest number of edges from one synset to another synset within the hierarchical WordNet structure, by also considering the depths of the two synsets in the WordNet hierarchy, along with the depth of the least common subsumer (lcs), as follows:

Wu–Palmer score = 2 × depth (lcs)/[depth (s1) + depth (s2)]

To perform this analysis, first, the audio from the reports was transcribed into plain text (^∗^.txt) files. All quantitative analyses were coded in Python and were carried out using the NLTK and WordNet. Next, the transcribed verbal reports were tokenized (i.e., parsed) using the NLTK into words for only the meaningful parts of speech, including: nouns, adjectives, verbs, and adverbs. After programmatically removing punctuation and stop-words (i.e., common words that do not contribute to the meaning of a sentence), each word was used to look up the corresponding synsets in the WordNet hierarchy database, thereby creating a list of synsets corresponding to the words in each verbal report. Note that a given word can belong to several synsets, and thus, all synsets for each word from the corpus of text were included in the Wu–Palmer Similarity analysis. Finally, the semantic distance (using Wu–Palmer Similarity) from each possible pair of synsets corresponding to a given word to another was calculated using the NLTK iteratively and exhaustively between pairs of synsets from the wake reports and the synsets of each dream report. This was also done between the wake reports and daydream reports. Then, an average of the pairwise Wu-Palmer calculations was calculated in order to measure the average (i.e., overall degree) of incorporation of the waking experience into the content of each set of dreams (early and late) and daydreams. In other words, this incorporation score reflects the average Wu Palmer similarity scores between all wake-dream report pairs of synsets. A score of 100 would represent complete identity between all the synsets from the wake report and all the synsets from the dream reports (n.b., a highly unlikely scenario), and zero would represent no semantic relationship between the synsets of the wake report and the synsets of the dream reports. For example, an incorporation score of 15 for early dreams, would mean that the average of all Wu–Palmer Similarity scores comparing synsets in the wake report to synsets in early dream reports was 15.

### Cognitive Abilities Testing

The CBS Trials battery (Hampshire et al., 2012) is composed of 12 online computerized cognitive tests that were designed based on well-established paradigms from the cognitive neuroscience literature (see: Hampshire et al., 2012) for detailed descriptions of the 12 individual tests). The CBS Trials can be used to assess a wide variety of cognitive abilities, which can be factored together to evaluate three higher-order cognitive domains described as “Verbal,” “Reasoning,” and “STM” subscales, derived from a data driven approach using factor analysis, conducted in a large population from a previous study (Hampshire et al., 2012). The Verbal subscale is composed primarily of verbal reasoning (Baddeley, 1968), color-word remapping (Stroop, 1935), and the digit span test (Wechsler, 1981). The Reasoning subscale is composed primarily of deductive reasoning (Cattell, 1940), spatial rotation (Silverman et al., 2000), feature match (Treisman and Gelade, 1980), spatial planning (Shallice, 1982), and the interlocking polygons test (Folstein et al., 1975). Lastly, the STM subscale is composed primarily of visuospatial working memory (Inoue and Matsuzawa, 2007), spatial span (Corsi and Micheal, 1972), paired associates (Gould and Brown, 2005), and the self-ordered search test (Collins et al., 1998). Raw scores from each of the 12 tests were normalized using the age matched population mean and standard deviation obtained from a large (*N* = 44,600) population (Hampshire et al., 2012). Next, to combine the subtests into their respective higher-order subscales, the normalized test scores were re-weighted using factor loadings from Hampshire et al. (2012), and then the respective tests which compose each factor-weighted subscale were averaged to create the Verbal, Reasoning, and STM subscales. Finally, the sub-test scores were transformed to a mean of 100 and a SD of 15 so that test scores were readily comparable to results from similar studies by our group and others that employed test batteries which assess reasoning and verbal abilities, such as the MAB-II (Fogel and Smith, 2006, 2011; Fogel et al., 2007a).

### Statistical Analyses

All statistical analyses were carried out using SPSS Statistics version 22 (IBM, Armonk, NY, United States). First, to confirm whether participants did in fact exhibit performance improvements after the nap retention period, paired *t*-tests were used to investigate performance improvements from the last two trials of the training session to the first two trials of the retest session. As well, independent *t*-tests were used to establish if there were any differences between the participants within the tennis and spatial navigation conditions in terms of the semantic similarity between their wake and dream reports and between their wake and daydream reports.

To systematically test the hypothesis of whether the relationship between dream incorporation and the extent of performance improvements (from training to retest, representing the extent of memory consolidation) were mediated by inter-individual differences in cognitive abilities, a well-established four-step mediation analysis procedure using multiple regression (as recommended by Judd and Kenny, 1981; James and Brett, 1984; Baron and Kenny, 1986) was employed. Step 1 regressed each dependent variable for performance improvement in separate analyses on the independent variables, dream incorporation (early, late, and daydreams), to test whether there was a significant relationship between dream incorporation and performance improvements. Step 2 regressed each mediator variable for dream incorporation (early, late, and daydreams) on the independent variables, cognitive abilities (Reasoning, Verbal, and STM), to confirm whether there was a significant relationship between dream incorporation and cognitive abilities. Step 3 regressed each dependent variable, for performance improvement in separate analyses on the mediator variables for cognitive abilities (Reasoning, Verbal, and STM) to determine whether there was a significant relationship between cognitive abilities and performance improvements. If Steps 1–3 were satisfied, then mediation was possible and would be tested in Step 4. If Step 4 was viable, each dependent variable for performance improvements was regressed in separate analyses on the mediator variables for cognitive abilities (Reasoning, Verbal, and STM) while controlling for dream incorporation (early, late, and daydreams) to determine whether the relationship in Step 1 was either partly or fully mediated by cognitive abilities. The purpose of Steps 1–3 was to establish that zero-order relationships existed among the various factors that may account for the relationship between dream incorporation and performance improvements. If one or more of these relationships were not statistically significant, then mediation was not possible, or statistically unlikely (Judd and Kenny, 1981; James and Brett, 1984; Baron and Kenny, 1986). All statistical results were considered significant at *p* < 0.05 for the multiple regression, and if significant, the results were further investigated by inspecting the partial coefficients to examine which of the independent variables uniquely accounted for variability in the dependent variable in the model.

## Results

### Performance Improvements

**FIGURE 4:**
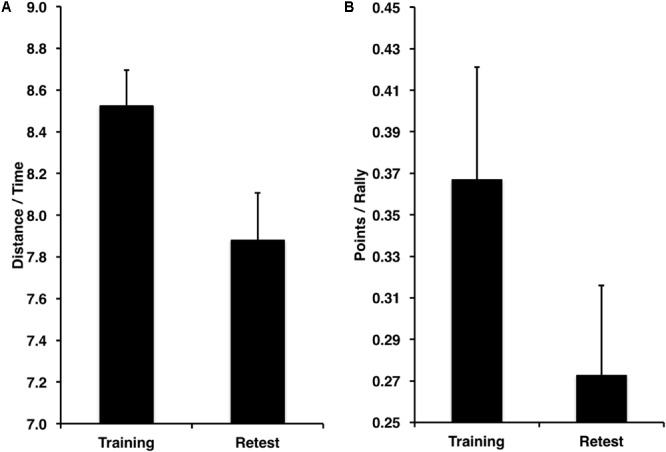
Significant performance improvements from training to retest in terms of speed for the **(A)** spatial navigation task in distance traveled toward the target (a.u.) per amount of time in seconds, **(B)** and number of points won per number of rallies played.

Performance improved from the training to the retest session for the spatial navigation task (**Figure 4A**) in terms of speed [distance/time from the start to the goal location; *t*(11) = 3.99, *p* < 0.001, *D* = 2.78]. Similarly, performance improved from training to retest for the tennis task (**Figure 4B**) in terms of points won per number of rallies played [*t*(11) = 2.13, *p* = 0.027, *D* = 2.07].

Given that participants were randomly assigned to the two task conditions and for the purposes of this study, there were no hypotheses about the task-specific nature of the incorporations, this study only assessed the relationship of the extent of the incorporation with performance improvements and also with cognitive abilities, irrespective of the particular daytime experience (e.g., tennis or spatial navigation), or lab environment. Thus, for or all subsequent analyses, unless stated otherwise, the tennis and spatial navigation conditions are considered together (*N* = 24).

### Incorporation of Waking Experiences Into Dreams and Daydreams

There was no difference between the spatial navigation (*N* = 12) and tennis (*N* = 12) conditions (**Table 1**) in terms of the extent of semantic similarity, reflecting incorporation, between the wake report and early dream reports [*t*(22) = 1.65, *p* = 0.113, *D* = 0.68], late dream reports [*t*(22) = 0.38, *p* = 0.705, *D* = 0.16], all dream reports combined [*t*(22) = 1.64, *p* = 0.115, *D* = 0.67], or daydream reports [*t*(22) = 0.44, *p* = 0.667, *D* = 0.22]. In addition, the magnitude of early dream incorporation did not differ from late dream incorporation [*t*(23) = 0.89, *p* = 0.382, *D* = 0.18]. Incorporation for early dreams [*t*(22) = 5.13, *p* < 0.001, *D* = 1.304], late dreams [*t*(22) = 3.59, *p* = 0.003, *D* = 0.91] and all dream reports combined [*t*(22) = 4.69, *p* < 0.001, *D* = 1.21] were significantly higher than daydream incorporation. Together, these results suggest that the extent of dream incorporation did not differ between the spatial navigation and tennis conditions, and that dream report content was more similar, in terms of extent of incorporation, than daydream content (see **Figure 2** for illustrative examples).

**Table 1:** Mean dream and daydream incorporation in the spatial navigation and tennis conditions and collapsed across conditions (all), where 0 = no semantic relationship and 100 = completely synonymous.

	Spatial navigation (*N* = 12)		Tennis (*N* = 12)		All (*N* = 24)	
	M	SD	M	SD	M	SD
Early	14.97	1.56	13.89	1.68	14.43	1.68
Late	14.30	1.32	14.07	1.65	14.18	1.47
Mean	14.77	1.35	13.77	1.63	14.27	1.55
Daydream	12.54	1.58	12.08	2.58	12.34^∗^	2.01

*^∗^Indicates significantly different from at *p* < 0.05 from early, late, and mean dream incorporation for all (*N* = 24 subjects).*

To assess the specificity of the incorporation, a supplementary analysis was conducted to measure the semantic similarity (between wake and dream reports) for each individual within a condition to all other individuals within the same condition, and another analysis to measure the semantic similarity for each individual to all other individuals in the other condition. Within-condition semantic similarity was higher than between-condition similarity for dreams in the tennis [*t*(11) = 5.56, *p* < 0.001, *D* = 1.61] and spatial navigation condition [*t*(11) = 3.98, *p* = 0.002, *D* = 1.15]. Suggesting that semantic relatedness was higher between individuals within the same condition than between individuals in different conditions. Thus, the extent of incorporation was not some epiphenomenon of relatedness between an individual’s wake report and their own subsequent dream (or daydream) report.

### The Relationship Between Dream Incorporation and Memory Performance, and Mediation by Inter-individual Differences in Cognitive Ability

#### Step 1: Dream Incorporation and Performance Improvements

**FIGURE 5:**
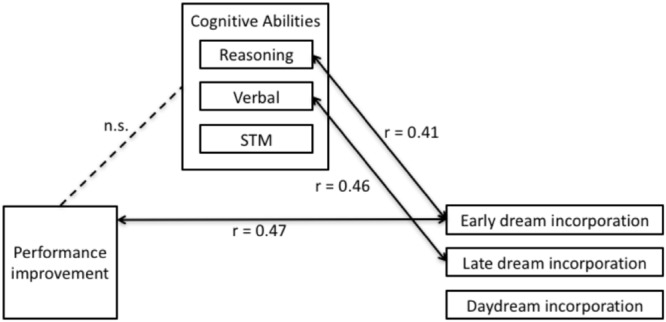
Inter-relationships between performance improvements, dream incorporation and cognitive abilities. Improved performance was correlated with early dream incorporation (*r* = 0.47), but not late dream incorporation or daydream incorporation. Early dream incorporation was directly correlated with reasoning (*r* = 0.41), but not verbal abilities or STM. Late dream incorporation was directly correlated with Verbal (*r* = 0.46) but not reasoning abilities or STM. There was no significant direct relationship between performance improvements and intellectual ability.

**FIGURE 6:**
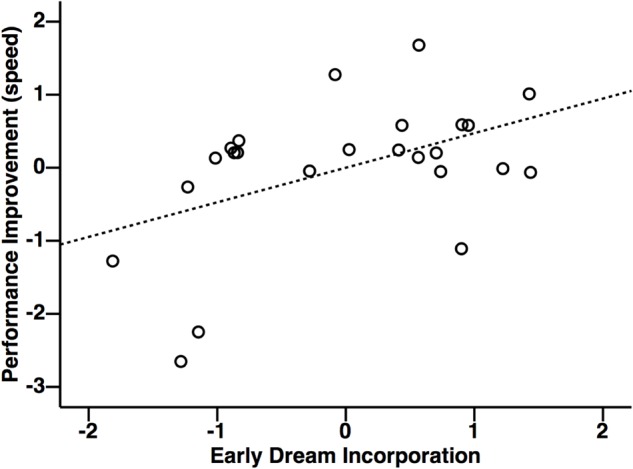
Relationship between performance improvements for the tennis task and early dream incorporation. Performance improvements were correlated with early dream incorporation, but not late dream incorporation, or daydream incorporation. Units for *x* and *y* axes are displayed in standardized residuals (Z) scores.

Multiple regression was used according to established procedures to test for mediation (Judd and Kenny, 1981; James and Brett, 1984; Baron and Kenny, 1986) to investigate the relationship between performance improvements with dream incorporation (**Figure 5**). This analysis revealed that early dream incorporation was correlated with performance improvements [*F*(2,21) = 3.60, *r* = 0.51, *p* = 0.045]. In this model, inspection of the partial coefficients revealed that this relationship was significant for the tennis task (**Figure 6**: *r* = 0.47, *p* = 0.023), but not for the navigation task (*r* = 0.08, *p* = 0.71). By contrast, performance improvements did not relate to late dream incorporation [*F*(2,21) = 1.68, *r* = 0.37, *p* = 0.21] or daydream incorporation [*F*(2,21) = 0.09, *r* = 0.12, *p* = 0.92].

In order to follow-up whether initial performance on the tasks was correlated with dream incorporation, the exact same analysis strategy was conducted using performance scores during the first two trials of the training session. None of the analyses revealed any statistically significant effects (all *p* > 0.90). Thus, suggesting that learning-related improvements, but not inter-individual differences in initial skill for the task, were related to the extent of dream incorporation for early, but not late dreams or daydreams.

#### Step 2: Dream Incorporation and Cognitive Abilities

**FIGURE 7:**
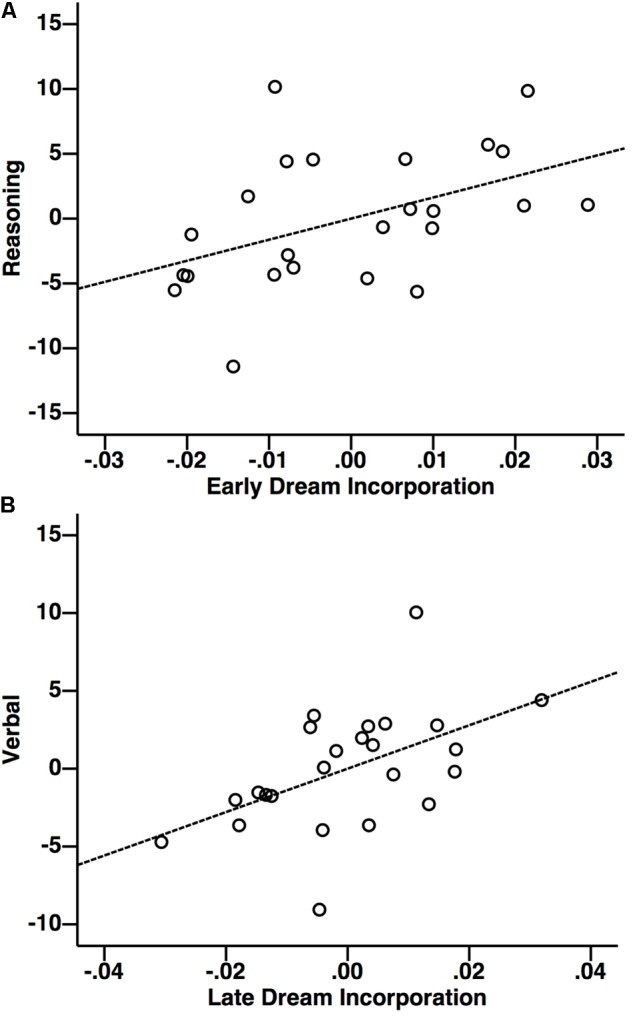
Relationship (partial correlation) between dream incorporation and reasoning **(A)** and with verbal **(B)** abilities. Greater early dream incorporation was associated with higher reasoning ability (controlling for verbal ability and STM), whereas greater late dream incorporation was associated with higher Verbal ability (controlling for reasoning ability and STM). Units for *x* and *y* axes are displayed in standardized residuals (Z) scores.

Multiple regression revealed that altogether Reasoning, Verbal, and STM accounted for a significant proportion of variability in early dream incorporation [**Figure 5**; *F*(3,21) = 4.17, *p* = 0.019]. Interestingly, in this model, inspection of the partial coefficients revealed that Reasoning (**Figure 7A**; *r* = 0.41, *t* = 2.36, *p* = 0.029), but not Verbal or STM significantly and uniquely accounted for variability in dream incorporation. Similarly, altogether Reasoning, Verbal, and STM accounted for a significant proportion of variability in late dream incorporation [**Figure 5**; *F*(3,21) = 3.18, *p* = 0.046). However, by contrast, inspection of the partial coefficients revealed that for late dream reports, Verbal (but not reasoning ability or STM) uniquely predicted late dream incorporation (**Figure 7B**; *r* = 0.50, *t* = 2.69, *p* = 0.014). Thus, suggesting a dissociation between cognitive abilities and early vs. late dream incorporation. In addition, there was no relationship between intellectual abilities and daydream incorporation [*F*(3,21) = 1.70, *p* = 0.1), suggesting that the relationship between cognitive abilities and incorporation may be specific to sleep and dreams.

#### Step 3: Cognitive Abilities and Performance Improvements

Multiple regression revealed that performance improvements were unrelated to cognitive abilities including Reasoning, Verbal, and STM (all *p* > 0.26). Given that Step 3 was not significant (**Figure 5**), according to Judd and Kenny (1981), James and Brett (1984), and Baron and Kenny (1986), this precludes the possibility that cognitive abilities mediate the relationship between performance improvements and dream incorporation, and thus Step 4 was not necessary to test for the nature or extent of the mediation (e.g., either total or partial mediation).

Finally, in order to follow-up whether initial performance on the tasks was correlated with cognitive abilities, the exact same analysis strategy was conducted using performance scores during the first two trials of the training session. Surprisingly, none of the analyses revealed any statistically significant effects (all *p* > 0.33).

## Discussion

Is there any rhyme or reason to the mental activity reflected in our dreams; and if so, what is the functional significance of these thoughts? Given the links between: (1) sleep and memory, (2) learning and dream incorporation, and (3) between sleep and cognitive abilities, we explored whether the richness of learning-related incorporation of novel daytime experiences into the content of our dreams was related to intellectual abilities. Similar to previous studies, it was found that improved performance, reflecting memory consolidation, was associated with the extent of dream incorporation into early but not late dream reports. Thus, suggesting that individuals who have richer dream incorporations, experience greater offline performance improvements. Surprisingly, the extent to which one improves on these tasks after sleep was unrelated to cognitive abilities, thereby precluding any mediation of the relationship between offline performance improvements and dream incorporation by inter-individual differences in cognitive abilities. In addition, inter-individual differences in the level of initial performance (first two trials of practice) at the training session did not relate to the extent of dream incorporation, or to inter-individual differences in cognitive abilities. Importantly, we identified a relationship between the extent of dream incorporation and reasoning abilities (but not verbal abilities or STM), and only for early dream reports. By contrast, verbal cognitive abilities were associated only with late dream reports. These effects appear to be specific to sleep, given that incorporation into daydreams was generally lower and unrelated to performance improvements, and also, unrelated to cognitive abilities. It is important to note that the association with cognitive abilities was investigated using dream incorporation of waking experiences, not dream frequency or dream content, *per se*. In the past, dream frequency and dream content were found to have only weak, inconsistent and disparate links to individual cognitive traits (Blagrove and Pace-schott, 2010). Taken together, these results suggest that individuals with greater performance improvements, more richly incorporate newly learned experiences into the content of their dreams, and that the extent of this dream incorporation was related to reasoning abilities. Thus, suggesting a trait-like relationship between the extent of the learning-related dream incorporation and cognitive abilities.

The idea that dreams may be linked to our cognitive capacities is not new (Foulkes, 1985; Domhoff, 2003, 2010). However, there is little empirical evidence supporting this relationship to date. The first scientific studies to suggest this link was in a series of longitudinal (Foulkes, 1982) and cross-sectional (Foulkes et al., 1990) studies in children exploring the relationship between REM dream content (e.g., dream frequency and narrative complexity) and cognitive capacities over the course of development in children age 5 to 15 years old. The only consistent predictor of dream frequency from the battery of cognitive aptitude tests (e.g., visuospatial, verbal, descriptive, and memory abilities), was the block design test of the WISC, which assesses visuospatial skills. Other studies also have shown that visual memory is associated with dream recall frequency (Cory et al., 1975; Butler and Watson, 1985; Schredl and Montasser, 1996a,b). Taken together, these studies suggest that dreaming is window into understanding how sleep relates to trait-like cognitive capacities. Surprisingly, to our knowledge, this is the first study in adults exploring the relationship between intellectual abilities and learning-related dream incorporation.

Not only have cognitive abilities been linked to dreams, they have also been linked to sleep-related memory consolidation. Specifically, Smith et al. (2004) found that individuals with high Intelligence Quotient (IQ) scores had greater learning-dependent changes in REMs during REM sleep. In addition, individuals with high Weschler Memory Scale scores had a greater declarative learning-related increase in sleep spindle activity, and benefitted more from sleep than individuals with lower scores (Schabus et al., 2006). However, Tucker and Fishbein (2009) found that IQ (as measured by the MAB-II) predicted better training and retest performance on declarative and procedural memory tasks, but did not correlate with overnight changes in performance. While the evidence remains sparse, together, these studies suggest that the features of REM and non-REM sleep, including REMs (Smith et al., 2004; Fogel et al., 2007a) and spindles (Schabus et al., 2006; Bódizs et al., 2008; Fogel and Smith, 2011; Geiger et al., 2011; Gruber et al., 2013; Hoedlmoser et al., 2014; Ujma et al., 2014; Fang et al., 2017), may serve as physiological indices of trait-like verbal and reasoning cognitive abilities, respectively. The results of the present study suggest that there may also be physiological correlates of reasoning abilities early (close to sleep onset) and for verbal abilities later in the sleep period during hypnagogic states. However, this possibility remains to be explored.

It is important to note that in the current investigation, while waking daydreams were collected, a separate wake control condition was not included, and thus, we cannot draw the conclusion that performance improvements were indeed “sleep-dependent,” *per se*. However, this was not the focus of the current investigation. Nonetheless, the performance improvements from training to retest support the notion that learning occurred, and that similar to previous studies, memory consolidation did take place (re; there was no forgetting, or performance decrements). Moreover, given that the relationship between improved performance and the extent of incorporation was only observed during early dreams and not daydreams would suggest that sleep was an opportune time for memory processing to take place. However, it should be noted that early dream incorporation was related only to performance improvement in the tennis but not the spatial navigation condition. In addition, dream incorporation was correlated to performance improvements for early but not late dream incorporation. This pattern of results warrants replication and further investigation in order to draw any meaningful conclusions or identify any important functional dissociations. Finally, while many of the *p*-values from the main statistical analyses were statistically significant, they did not greatly exceed *p* < 0.05. However, we did have strong *a priori* hypotheses, which flowed from the previous literature, and tested these hypotheses using a systematic approach to look for possible mediation of the three main sets of variables (dream incorporation, cognitive abilities, and memory performance). This was done using multiple regression, following the very well established procedures proposed by Judd and Kenny (1981), James and Brett (1984), and Baron and Kenny (1986). Importantly, it should be noted that the analysis procedure employed here has relatively low power, and thus, detection of statistical significance may be underestimated. Indeed, upon inspection of the partial coefficients, statistically significant effect sizes ranged from 0.41 to 0.48, which according to Cohen’s standards (Cohen, 1988) are considered “medium” effect sizes (>0.3). Moreover, Kenny points out that because these are indirect effects, and the product of two effects, they actually underestimate effect sizes, arguing instead that Cohen’s recommendations should be squared. If so, our results should all be considered to have “large” effect sizes. In other words, in contrast to previous studies, our main findings explain over 40% of the variability in our variables of interest (e.g., dream incorporation).

The capacity for reasoning, as assessed in the present study by the CBS Trials test battery, tap into aspects of “fluid intelligence” (adapted from items from the Cattell Culture Fair Intelligence Test), which most closely relate to the WAIS (and MAB-II) Performance IQ, and the Raven’s Advanced Progressive Matrices. Fluid intelligence has been said to represent the innate (i.e., constitutionally and biologically endowed) intellectual potential that gives rise to, but is distinct from crystalized intelligence, which develops through learning and experience (Boyle, 1988). It is considered the capacity to identify complex patterns and relationships, and the capacity to employ logic to solve novel problems. The relationship between reasoning abilities and early dream incorporation may reflect individual differences in how information is processed during sleep. The nature and the extent of this processing may depend on our reasoning abilities. When new information is learned, sleep is involved in the reactivation (Peigneux et al., 2003, 2006; Bergmann et al., 2011; Antony et al., 2012; Oudiette and Paller, 2013; Oudiette et al., 2013; Schönauer et al., 2014; Fogel et al., 2017), and even replaying (Skaggs and McNaughton, 1996; Louie and Wilson, 2001; Lansink et al., 2009) of newly learned experiences. This memory reprocessing is thought to be reflected in the content of our dreams as incorporations of waking novel, immersive or impactful experiences (Stickgold et al., 2000; Wamsley et al., 2010a,b; Kusse et al., 2012; Wamsley, 2014). These incorporations are not 1:1 representations of past experiences, rather, they tend to be intermingled with other seemingly unrelated content. This might reflect the process of integrating and transforming this new information into existing memory representations. Moreover, sleep is involved in facilitating the transformation of unconscious knowledge into conscious knowledge and problem solving (Wagner et al., 2004; Darsaud et al., 2011; Verleger et al., 2013; Spiers and Bendor, 2014). Here, the present study suggests that the extent of this processing might depend on reasoning abilities; the capacity to solve novel problems through the use of logic.

Given that indirect incorporations tend to occur later in the sleep period (Wamsley et al., 2010a) and that later dreams contain more indirect than direct incorporations (Wamsley et al., 2010a), it is possible that detecting a relationship with intellectual abilities is more difficult during late dreams due to the sparsity and disparate semantic links to waking experiences. This is, however, not necessarily supported by the present results given that Verbal abilities were significantly correlated with late dream incorporation. One parsimonious and interesting interpretation of this result is that individuals with better Verbal cognitive abilities are able to access, recall and articulate these more disparate dream incorporations more robustly. On the other hand, these individuals may be able to more richly recall dream content in general, and thus increase the probability of reporting related concepts. This may also explain why a difference in the absolute extent of incorporation between early and late dream reports was not observed. However, this interpretation remains speculative, and may require further study. There is, however, evidence to support the notion that high vs. low dream recallers have longer awakenings in response to auditory stimuli during sleep, regardless of sleep stage (Vallat et al., 2017), and greater brain reactivity regardless of sleep or wake (Eichenlaub et al., 2014). Thus, suggesting that high dream recallers likely process information to a greater extent than low dream recallers during sleep, and are more easily aroused. This may partly explain inter-individual variations in dream recall. Together, these results help to elucidate the relationship between verbal abilities and late dream incorporation – when dream incorporation is less directly semantically related, and possibly more difficult to recall and articulate. Future studies could investigate the neural correlates of the relationship between dream incorporation and cognitive abilities. Moreover, given the specific focus of the current study on non-REM dream content, we cannot infer, or directly compare these findings to REM dream-related phenomena. Interestingly, however, learning-related dream incorporation into REM dreams is strongest on the night after and from 5–7 nights after learning, reflecting “day residue” and “dream-lag” effects, respectively (Nielsen et al., 2004; van Rijn et al., 2015). It would be important for future studies to apply the approach developed here to study the relationship between cognitive abilities and learning-related incorporation into REM dream content, when dream incorporation is maximal.

The relationship between cognitive abilities and dream incorporation appeared to be specific to sleep and also specifically related to the experimental learning conditions. There was no relationship between behavioral improvement and incorporation into daydream reports, nor any relationship between daydream incorporation and cognitive abilities. Thus suggesting that the relationship between dream incorporation and cognitive abilities are specific to sleep, and not mental content during wakefulness interspersed between sleep episodes, i.e., “daydreaming.” Moreover, within condition semantic similarity was higher than between conditions. Thus, the overall pattern of results was specific to sleep and to the unique incorporation of the learning-related experience.

A major challenge to the study of dreams is that they are typically analyzed by rating and ranking the content of verbal dream reports along various dimensions by judges (Hall and Van De Castle, 1966), or by the dreamers themselves. With the exception of a few recent studies (Amini et al., 2011; Horikawa et al., 2013; Wong et al., 2016), the majority of dream research has been limited to the study of subjectively scored and interpreted dream reports. However, recent advances in language processing and machine learning techniques (Amini et al., 2011; Horikawa et al., 2013; Wong et al., 2016) have made the objective analysis of dream reports possible. The current study employed WordNet, a manually curated publicly available lexical database of the English language that can be used to derive the high-order meaning of words from a corpus of text, as well as the semantic distance between the higher-order senses of words with another corpus of text. In this way, we were able to objectively derive the higher-order semantic meaning of the wake reports generated immediately after mental rehearsal of the previously learned task. We could then compute the semantic distance between these concepts with subsequent dream reports obtained from post-learning sleep. This method is advantageous in that it is not necessary for a human scorer to interpret the meaning of the words in the reports, or to infer how direct incorporations were. Moreover, this approach avoids the possibility that individuals with higher cognitive abilities may be able to better generate confabulations about waking life into dreams (Henley-Einion and Blagrove, 2014). This provides a novel method to objectively quantify dream incorporation for future studies.

Ultimately, this line of investigation leads to the question: can dreaming actively contribute to intellectual functioning and cognitive abilities? Unfortunately, despite the use of mediation analysis, the direction of causation cannot be inferred from the results of this study, as these relationships are inherently correlational. However, the present study suggests that when something new is learned and this new learning is incorporated into dreams, the extent to which this occurs is associated with improved performance, and also with the capacity for reasoning. This is consistent with the proposition that cognitive abilities support dream formation (De Koninck, 2012) and that dreaming is a period where the brain, being unfettered by the constraints of information processing during waking (Klinger, 1978), is ideal for novel and creative thought (Foulkes, 1985) that may support novel solutions to new problems (Barrett, 1993) and creative thinking (Bone and Corlett, 1968; Fitch and Armitage, 1989; Streich, 2009). To the best of our knowledge, there are no studies to date that have investigated whether dream incorporation is related to cognitive abilities. One study, however, by De Koninck et al. (1989) found that during a period of intensive second language learning, those who progressed well, experienced incorporations into dreams earlier and had more verbal communication in their dreams during the language training than those who made little progress. Taken together, these studies are consistent with the conclusion that there is a relationship between learning proficiency and cognitive abilities during dreaming in humans.

In summary, here we identified, for the first time, that the extent of dream incorporation of a novel and immersive period of learning experience was related to cognitive abilities. These reflect the ability to apply logic and reasoning to solve problems (or, what is also known as Performance IQ, or fluid intelligence). The relationship between dream incorporation and reasoning abilities was strongest in early dream reports, when incorporations have been found to be more direct (Wamsley et al., 2010a), and when offline improvements in performance were related to the extent of the dream incorporation. On the other hand, there was no relationship with early dream reports to other cognitive abilities that reflect the use of existing knowledge (also known as Verbal IQ or crystalized intelligence), or to STM. However, the extent of dream incorporation was related to verbal abilities for dream reports collected later in the sleep episode when incorporations have been found to be less direct (Wamsley et al., 2010a). This finding suggests that individuals with greater verbal abilities are able to better recall or articulate weak incorporations.

These results provide evidence that the extent to which we incorporate newly learned material into our dreams is related to our cognitive abilities, in particular, our capacity for reasoning, problem solving and the use of logic. On the other hand, while verbal abilities are related to dream incorporation, it is at a time where incorporations are disparate, and when dream incorporation is unrelated to memory consolidation. Importantly, this study provides evidence that dream production is related to our cognitive strengths and weaknesses.

## Author Contributions

LR and VS carried out the study. SF and AO were involved in planning and supervised the work. LR and SF processed the data, performed the analysis, drafted the manuscript, and designed the figures. JDK aided in interpreting the results and worked on the manuscript. All authors discussed the results and commented on the manuscript.

## Conflict of Interest Statement

The authors declare that the research was conducted in the absence of any commercial or financial relationships that could be construed as a potential conflict of interest.
